# Analysis of Maternal and Neonatal Complications in a Group of Patients with Gestational Diabetes Mellitus

**DOI:** 10.3390/medicina57111170

**Published:** 2021-10-28

**Authors:** Agnesa Preda, Vlad Pădureanu, Maria Moța, Adela-Gabriela Ștefan, Alexandru Cristian Comănescu, Lucrețiu Radu, Emilia Roxana Mazilu, Ionela Mihaela Vladu

**Affiliations:** 1 Department of Obstetrics and Ginecology, Clinical County Emergency Hospital, 200349 Craiova, Romania; agnesapcela@yahoo.com (A.P.); rdtroxana@gmail.com (E.R.M.); 2 Department of Internal Medicine, Faculty of Medicine, University of Medicine and Pharmacy of Craiova, 200349 Craiova, Romania; 3 Department of Diabetes Nutrition and Metabolic Diseases, Faculty of Medicine, University of Medicine and Pharmacy of Craiova, 200349 Craiova, Romania; mmota53@yahoo.com (M.M.); mihmitzu@yahoo.com (I.M.V.); 4 Department of Diabetes Nutrition and Metabolic Diseases, Calafat Municipal Hospital, 205200 Calafat, Romania; adela.firanescu@yahoo.com; 5 Department of Obstetrics and Ginecology, Faculty of Medicine, University of Medicine and Pharmacy of Craiova, 200349 Craiova, Romania; alecom8000@yahoo.com; 6 Department of Hygiene, Faculty of Medicine, University of Medicine and Pharmacy of Craiova, 200349 Craiova, Romania; lucretiu.radu@gmail.com

**Keywords:** pregnancy, gestational diabetes mellitus, maternal complications, neonatal complications

## Abstract

*Background and Objectives*: Gestational diabetes mellitus (GDM) represents one of the most common complications during pregnancy, being associated with numerous maternal and neonatal complications. The study aimed to analyze maternal and neonatal complications associated with GDM. The risk factors of GDM and of the maternal and neonatal complications were studied in order to prevent their occurrence. *Materials and Methods*: The study included 97 women in the study, who underwent an oral glucose tolerance test (OGTT) between weeks 24–28 of pregnancy, consequently being divided into two groups: pregnant women with and without GDM. Statistical analysis was performed using the SPSS 26.0 software and MATLAB fitglm, the results being considered statistically significant if *p* < 0.05. *Results*: We observed statistically significant differences between the group of women with and without GDM, regarding gestational hypertension (17.6% vs. 0%), preeclampsia (13.72% vs. 0%), and cesarean delivery (96.1% vs. 78,3%). Data on the newborn and neonatal complications: statistically significant differences were recorded between the two groups (GDM vs. no GDM) regarding the average weight at birth (3339.41 ± 658.12 g vs. 3122.83 ± 173.67 g), presence of large for gestational age (21.6% vs. 0%), macrosomia (13.7% vs. 0%), excessive fetal growth (35.3% vs. 0%), respiratory distress (31.4% vs. 0%), hospitalization for at least 24 h in the Neonatal Intensive Care Unit (9.80% vs. 0%), and APGAR score <7 both 1 and 5 min following birth (7.8% vs. 0%). Additionally, the frequency of neonatal hypoglycemia and hyperbilirubinemia was higher among newborns from mothers with GDM. *Conclusions:* The screening and diagnosis of GDM is vital, and appropriate management is required for the prevention of maternal and neonatal complications associated with GDM. It is also important to know the risk factors for GDM and attempt to prevent their appearance.

## 1. Introduction

According to the American Diabetes Association (ADA), gestational diabetes mellitus (GDM) is defined as “a previously unknown diabetes, diagnosed during the second or third pregnancy trimester” [1]. The prevalence of GDM has significantly increased in the last 20 years [2].

GDM represents one of the most common complications during pregnancy [3,4]. According to the International Diabetes Federation (IDF) [3,5], the epidemiology of GDM is unknown in many countries due to the differences in diagnosis criteria [3,6]. Still, the GDM prevalence has reached alarming rates worldwide, increasing by more than 35% in the last few decades [3,4].

GDM is associated with both maternal and neonatal complications, such as preeclampsia, macrosomia, premature birth, hypoxia, and hypoglycemia [3,7,8]. Pregnant women with GDM have a higher risk of giving birth to large children for gestational age (LGA—a birth weight ≥ than the 90th percentile for a given gestational age or ±2 SD from the normal average for gestational age, sex, and race) [3]. Moreover, these women are prone to deliver by caesarean section, and the newborn may suffer injuries during birth [3]. GDM is also associated with an increased risk of neonatal respiratory complications and hospitalization within the Neonatal Intensive Care Unit (NICU) [8].

In our study, we analyzed maternal and neonatal complications in a group of pregnant Romanian women with and without GDM. At the same time, the risk factors of GDM were analyzed, so as to prevent its occurrence by early intervention on the modifiable risk factors.

## 2. Materials and Methods

### 2.1. Participants

We performed an epidemiological, prospective, non-interventional study over a period of 2 and a half years (December 2018–April 2021). We included 97 pregnant women in the study, who were monitored during the pregnancy and on whom we performed an oral glucose tolerance test (OGTT) with 75 g anhydrous glucose between weeks 24 and 28 of pregnancy. According to the obtained results, the pregnant women were divided into 2 groups: group 1–51 pregnant women with GDM; group 2–46 pregnant women without GDM.

Inclusion criteria: age over 18 years old, pregnant women who signed the study’s informed consent form and were monitored during pregnancy in the Emergency County Hospital, Craiova or Filantropia City Hospital, Craiova.

Exclusion criteria: women with type 1 and 2 DM prior to pregnancy, pregnant women aged less than 18 years, presence of other severe comorbidities (renal and cardiovascular diseases, anemia, thyroid disorders, cancer, etc.) or ongoing treatment that may influence the maternal and perinatal outcome, women who delivered outside the Emergency County Hospital, Craiova and the Filantropia City Hospital, Craiova, and women who did not show up for follow-up visits. In order to exclude overt diabetes, at the first prenatal visit of the pregnant women, we performed fasting blood glucose and we confirmed the diagnosis of diabetes mellitus when the blood glucose was repeatedly ≥126 mg/dL; we did not use for diagnosis the value of HbA1c ≥6.5%, knowing that possible errors can occur during pregnancy [9]. Currently, in many European countries, including Romania, selective screening is carried out at 24–28 weeks of gestation, based on the risk factors identified in the first trimester; thus, 30–50% of GDM cases can be lost. An increasing number of professional societies have adopted universal screening, even if there is a suspicion of overestimating the number of cases and increasing the cost. In the end, the cost/efficiency ratio is favorable, avoiding a series of early maternal and fetal complications, but also late ones, related to the mother and the child [10,11]. No international consensus has yet been reached on the type of GDM screening. Additionally, an increasing number of studies show that there are no significant differences between the incidence of GDM cases detected by the two methods: “the one step” approach with 75 g oral glucose tolerance (OGTT) (The International Association of Diabetes and Pregnancy Study Groups (IADPSG) recommendations, based on HAPO study) and “the two step” approach with 50 g nonfasting glucose screening, followed by 100 g OGTT for the patients who screen positive; in addition, the one-step approach is easier for patients to accept but also less expansive [10].

The subject participation was voluntary, and all pregnant women signed the informed consent form. The study was performed according to the ethical principles of the updated Helsinki Declaration, according to Good Clinical Practice (GCP) guidelines, complying with the right of integrity, confidentiality, and giving the subject the option to withdraw from the study at any moment.

The data of every participant in the study included: demographic data, personal physiological history (number of previous gestations, number of miscarriages, number of interrupted pregnancies, history of in utero fetal death, fetal macrosomia), anthropometric data immediately before pregnancy (height, weight, the body mass index—BMI), and personal and familial history. Pregnancies were considered stopped in evolution if the fetus death occurred before the gestational age of 20 weeks. Subsequently to this age, the death was considered fetal death in utero. An unfavorable obstetrical medical history was represented by repeated miscarriages, fetal death in utero, and the presence of fetal malformations [12].

Gestational age was determined by ultrasound and by calculating the duration from the first day of the last menstruation. The BMI (weight, in kilograms/square height, in meters) was classified according to the World Health Organization (WHO) [13]. Blood pressure (BP) was measured using an automatic sphygmomanometer in subjects in a sitting position after 10 min of rest. Pregnant women were considered hypertensive if they presented systolic BP (sBP) values ≥140 mmHg and/or diastolic BP (dBP) ≥90 mmHg and/or anti-hypertensive treatment at home. Gestational hypertension was considered a high BP diagnosed after 20 weeks of amenorrhea.

Regarding the age of pregnancy at delivery time, this was divided into the following categories: premature birth (24–37 weeks of gestation), on-term birth (38–42 weeks of gestation), and postmature birth (over 42 weeks of gestation).

The fetus was considered to be adequate for gestational age (AGA) if its birth weight was between the 10th and 90th percentiles. If the weight was below the 10th percentile, the newborn was considered small for gestational age (SGA). Fetuses were considered large for gestational Age (LGA) if they presented a birth weight above the 90th percentile. Macrosomia was considered for fetuses with a birth weight over 4000 g, regardless of the gestational age. Excessive fetal growth has been defined as LGA plus macrosomia.

### 2.2. Laboratory Tests

Laboratory tests were represented by fasting plasma glucose (FPG) after the first prenatal visit, followed by the oral glucose tolerance test (OGTT), with 3 determinations of blood glucose values: fasting, one hour, and two hours after loading with 75 g anhydrous glucose; another OGTT was performed postpartum in women with GDM in order to exclude type 2 DM occurring during pregnancy. The samples were obtained after overnight fasting (8–12 h). First, prenatal blood glucose and OGTT values performed at 4–12 weeks postpartum were interpreted according to standard diagnostic criteria [1].

The GDM diagnosis was established according to IADPSG (Table 1) [14].

Additionally, following the neonatologist’s recommendation, the newborn’s blood sugar and total bilirubinemia were collected after birth. We considered neonatal hypoglycemia the value of glycemia <40 mg/dL measured 0–2 h after birth and <45 mg/dL in the case of glycemia measured after 3–47 h and 48–72 h, respectively [15]. Hiperbilirubinemia, taking into account the physiological jaundice, was defined as the value of total bilirubinemia >13 mg/dL [16].

### 2.3. Statistic Analysis

The data were recorded in the EXCEL database and then transferred to the Statistical Package for the Social Sciences (SPSS) 26.0 (SPSS, Inc., Chicago, IL, USA), coded, and analyzed with this software. All data were analyzed according to the presence or absence of GDM in pregnant women included in the study.

The distributions of the continuous variables were tested for normal values by using the Kolmogorov–Smirnov test. Data with a normal distribution were presented as a mean value ± standard deviation (SD); the data that did not have a normal distribution were presented as a median and interquartile rate (IQR). To determine the statistical significance of the differences between the two groups, we used the Student’s *t*-test for comparing the means and the Mann–Whitney U test, respectively, for comparing the medians. The percentages between the two groups were compared using the chi-squared test. We used also MATLAB fitglm for logistic regression analysis. All the performed tests were considered statistically significant if they recorded a *p* value < 0.05.

## 3. Results

The general characteristics of pregnant women included in the study are presented in Table 2. Pregnant women with GDM had a statistically significant higher age at birth (*p* < 0.001) compared to the control group, showed a statistically significant higher percentage of BMI ≥ 30 kg/m^2^ (*p* = 0.029) before pregnancy, significantly increased in weight during pregnancy (*p* = 0.046), and showed a higher percentage of GDM in previous pregnancies (*p* = 0.05). Unfavorable obstetrical history, as well as fetal macrosomia in previous pregnancies, was exclusively presented in the group of pregnant women with GDM, and the difference was highly statistically significant. Highly statistically significant differences were also recorded between the groups (*p* < 0.001) regarding FPG at first prenatal visit, but also regarding OGTT, with the glycemic value being higher in pregnant women with GDM.

We performed univariate logistic regression analysis of GDM for each clinical characteristic as well as a multivariate logistic regression analysis in order to detect the independent risk factors of GDM. The following factors were statistically significantly correlated with the presence of GDM: age (*p* = 0.0001), macrosomia in previous pregnancies (*p* = 0.002), bad obstetric history (*p* = 0.009), previous GDM (*p* = 0.021), and weight gain during pregnancy (*p* = 0.04). By multivariate logistic regression analysis: none of the risk factors for GDM were significantly correlated with the presence of GDM, after adjusting for the other risk factors, reconfirming the multifactorial etiology of GDM.

By analyzing maternal complications, gestational hypertension was found in a higher percentage in the group with GDM (17.6%), unlike the control group, where there was no case recorded (0%); a statistically significant difference (*p* = 0.003). Regarding preeclampsia, it was strictly found in the group with GDM (13.72%), a highly statistically significant difference (*p* < 0.001) (Table 3).

Most pregnant women with GDM required a cesarean section for delivery (96.1%), vs. 78.3% in the control group, and the difference was statistically significant (*p* = 0.008) (Table 3).

We performed univariate logistic regression analysis of the data from Table 3, and we found that all of the outcomes (considered as dependent variables) correlated statistically significantly with the presence of GDM (potential risk factor for such outcomes, as an independent factor): gestational hypertension (*p* = 0.0001), preeclampsia (*p* = 0.002), vaginal delivery (*p* = 0.005), and emergency cesarean section (*p* = 0.005).

Regarding neonatal complications, prematurity was found in a higher percentage in the group with GDM (17.6%), vs. the control group (13%); still, the difference did not reach statistical significance. The duration of pregnancy was longer in the control group vs. the group with GDM (*p* = 0.097), still without reaching statistical significance. Macrosomia was strictly found among pregnant women with GDM (13.7%), with a statistically significant *p* value (0.009). Excessive fetal growth (LGL plus fetal macrosomia) was found with a higher statistical significance in the group with GDM vs. the control group (*p* < 0.001). The newborn weight was higher in the group of pregnant women with GDM compared to the control group, with a statistically significant difference (0.027). The association between GDM and SGA was strictly found in the pregnant women with GDM (5.9%), without reaching a statistically significant difference (*p* = 0.095). Highly statistically significant differences were found regarding AGA (*p* < 0.001); all the newborns in the control group (100%) were included in this category vs. 58.8% in the group with GDM (Table 4). Respiratory distress was found exclusively in newborns delivered by mothers with GDM (31.4%), the difference being highly statistically significant (*p* < 0.001). Additionally, a statistically significant difference (*p* < 0.001) was recorded regarding hospitalization for at least 24 h in NICU, found only among the newborns delivered by mothers with GDM. The APGAR score, evaluated both 1 and 5 min after birth, recorded the value <7 exclusively in newborns from mothers with GDM (7.8%), a difference at the limit of statistical significance (*p* = 0.05). Hypoglycemia, as a neonatal complication, was found exclusively in the newborns from mothers with GDM. Congenital abnormalities (hypertrophic cardiomyopathy) were found in a single newborn belonging to the group of pregnant women with GDM (2%).We performed univariate logistic regression analysis of neonatal complications (considering them dependent variables) and found that some of them are statistically significantly more common in children of GDM women: macrosomia (*p* = 0.002), APGAR score <7 (*p* = 0.002), hospitalization ≥ 24 h in NICU (*p* = 0.009), and SGA (*p* = 0.04).

## 4. Discussion

GDM is known as a risk factor for the onset of maternal and neonatal complications. In our study, we highlighted the impact of GDM both on the fetus and the mother. Thus, we recorded statistically significant differences regarding gestational hypertension, but also of preeclampsia. Additionally, pregnant women with GDM required cesarean section in a larger number than the ones in the control group. These data are in accordance with the literature [3,8]. In our patients, cesarean section rates are very high (78.3% in the group without GDM), which is higher than the WHO forecast for 2030 [17]. Moore et al. analyzed the differences in cultural values surrounding cesarean birth, suggesting that a reduction in this mode of birth would be possible [18]; Walid D et al. reported significantly higher total cesarean rates in diabetic women (50.2%) but much lower than in our group of patients [19]. Other authors consider that the main indication for cesarean delivery would be fetal risk, primarily caused by chronic exposure to glycemic variability [20,21]. In Romania, increasing attention has been paid in recent years, to the decrease in stillbirths, both during the evolution of the pregnancy and during the birth. In our study, the number of stillbirths was zero, including in pregnant women with GDM, although the risk factors involved in increasing stillbirths were present in high percentages in women with GDD: intrauterine growth restriction 5.9%, total hypertension (chronic hypertension and gestational hypertension) 27.45%, preeclampsia 13.2%, bad obstetric history 9.8%.

Analysis of data from Latvia (the Medical Birth Register on stillbirth), a retrospective cohort study (2001–2014), showed that the stillbirth rate was 6.2 per 1000 births. In England, the stillbirth rate was 4.2 per 1000, which is lower than in Latvia [22,23].

The percentage of women with GDM who underwent emergency cesarean section in our study was 96.1%, and the main causes were fetal, maternal–fetal, or maternal suffering. Although the results are good, with zero mortality for both the fetus and the mother, it raises the question of whether this high percentage of emergency cesarean birth is also based on cultural reasons. Large glycemic variability during pregnancy frequently induces fetal distress and can often indicate cesarean section. There is a need for a multidisciplinary approach to pregnant women with risk factors, and an important consideration is a team that includes a diabetologist, cardiologist, and often a nephrologist, as other researchers claim [24].

As in other recent publications [2,3], in our study, prematurity was found more frequently as a neonatal complication in the group of women with GDM, but without statistical difference. The higher rate of preterm birth in GDM women could be explained by several factors; the most important appear to be arterial hypertension and preeclampsia [21,25].

Both fetal macrosomia and excessive fetal growth were found in the newborns delivered from mothers with GDM. These complications are frequently cited in the literature by other authors and are correlated with the presence of GDM [2,3]. Intermittent hyperglycemia (with normal HbA1c) appears to play a larger role than chronic hyperglycemia (usually associated with higher HbA1c), as has been shown in several studies [21,26].

Respiratory distress, APGAR score < 7, and the necessity for at least 24 h hospitalization in the NICU were found more frequently in the newborns from mothers with GDM in our study, which correlates with the data provided by other studies [2,8].

Neonatal hypoglycemia and congenital abnormalities were found more frequently in the newborns delivered by mothers with GDM, compared to the control group, data that coincide with the specialty literature [2,8].

Although prematurity, neonatal hypoglycemia, and congenital abnormalities were found more frequently in the group of pregnant women with GDM, statistical significance was not reached due to the low number of pregnant women enrolled in the study, and the lack of constant monitorization of the newborn glycemia, outside of the clinical symptoms.

The present study includes the following limitations: observational studies, generally, may suffer from the effect of bias, with patient selection being the most important. In our study, this bias was overcome, as the centers that enrolled the patients in the study were limited and applied the same protocol; because HbA1c was not performed periodically in all pregnant women, it was not possible to analyze the outcomes according to glycemic balance, although all women with GDM were permanently monitored by teams of diabetologists from the two hospitals. The aim was to reach the target values of glycemia, according to ADA standards of medical care in diabetes: fasting glycemia < 95 mg/dL, 1 h postprandial glycemia < 140 mg/dL and at 2 h postprandial glycemia < 120 mg/dL. Only 7.8% of women needed drug treatment; the remaining 92.2% of women reached the target values only with diet and physical activity. The diabetologist and/or gynecologist were able to persuade the pregnant women with GDM to perform postpartum OGTT with 75 g of glucose in order to rule out possible type 2 diabetes that occurred during pregnancy. The relatively small number of patients studied, mainly due to the absence of blood glucose before week 24 or the impossibility of postpartum metabolic evaluation, limited the statistical analysis of the data.

## 5. Conclusions

GDM is associated with accelerated fetal growth and certain adverse maternal and neonatal outcomes. In summary, the vital importance of GDM screening and diagnosis emerges, appropriate management being necessary to prevent the maternal and neonatal complications with which it is associated. Diagnosing GDM helps to evaluate the risk for GDM in subsequent pregnancies and also the cardio-metabolic risk for the mother and child during their lifetime, requiring primary prevention for this pathology, both in mother and child.

## Figures and Tables

**Table 1:** Diagnosis criteria for GDM (OGTT with 75 g anhydrous glucose).

GDM	Fasting Glycemia	1 h Glycemia	2 h Glycemia	Observations
	≥92 mg/dL (5.1 mmol/L)	≥180 mg/dL (10 mmol/L)	≥153 mg/dL (8.5 mmol/L)	A single pathological value may support the diagnosis

GDM, gestational diabetes mellitus.

**Table 2:** Clinical characteristics of the women with GDM and control group.

Characteristics		GDM Women	Control	*p*
Average age in years (SD)		32.39 ± 4.66	28.61 ± 4.71	<0.001
Median gravidity		1	0	0.355
Gestational age at delivery (weeks)		37.73 ± 2.07	38.35 ± 1.50	0.097
Maternal age over 40		1 (2%)	2 (4.3%)	0.498
BMI ≥ 30 kg/m^2^, before pregnancy		5 (9.8%)	0 (0%)	0.029
BMI (kg/m^2^)		22.96 ± 3.44	22.75 ± 2.60	0.734
Height (cm)		165.06 ± 4.99	163.00 ± 7.49	0.120
Macrosomia in previous pregnancies		7 (13.7%)	0 (0%)	0.009
Previous GDM		4 (7.8%)	0 (0%)	0.05
First-degree relative with diabetes		17 (33.3%)	8 (17.4%)	0.073
Bad obstetric history *		5 (9.8%)	0 (0%)	0.029
Weight gain during pregnancy (kg)		14.61 ± 4.47	12.48 ± 5.87	0.046
Fasting plasma glucose at first prenatal visit (mg/dL)		79.37 ± 9.34	71.39 ± 9.16	<0.001
OGTT	Fasting plasma glucose (mg/dL)	86.55 ± 21.63	72.74 ± 10.08	<0.001
	1 h plasma glucose (mg/dL)	176.71 ± 18.48	123.13 ± 23.53	<0.001
	2 h plasma glucose (mg/dL)	145.84 ± 19.33	107.09 ± 20.35	<0.001
Treatment for GDM	Diet	51 (100%)	0 (0%)	
	Insulin	4 (7.8%)	0 (0%)	
	OAD	0 (0%)	0 (0%)	

* Repeated miscarriages, fetal death in uterus, presence of fetal malformation. GDM, gestational diabetes mellitus; BMI, body mass index; OGTT, oral glucose tolerance test; OAD, oral antidiabetic drugs.

**Table 3:** Maternal complications associated with GDM.

Characteristics			GDM Women	Control	*p*
Hypertension		Total	14 (27.45%)	0 (0%)	<0.001
		Gestational	9 (17.6%)	0 (0%)	0.003
		Pre-existing hypertension	5 (9.8%)	0 (0%)	0.029
Preeclampsia			7 (13.72%)	0 (0%)	<0.05
Type of delivery	Vaginal delivery		2 (3.9%)	10 (21.7%)	0.008
	Cesarean section	Total	49 (96.1%)	36 (78.3%)	0.008
		Elective cesarean section	0 (0%)	0 (0%)	-
		Emergency cesarean section	49 (96.1%)	36 (78.3%)	0.008

GDM, gestational diabetes mellitus.

**Table 4:** Neonatal complications associated with GDM.

Characteristics		GDM Women	Control	*p*
Birth	Term birth	42 (82.4%)	40 (87%)	0.531
	Prematurity	9 (17.6%)	6 (13%)	
	Overterm birth	0 (0%)	0 (0%)	
	Average gestational age at birth (weeks)	37.73 ± 2.07	38.35 ± 1.50	0.097
Birthweight	Average birth weight (g)	3339.41 ± 658.12	3122.83 ± 173.67	0.027
	Excessive fetal growth	18 (35.3%)	0 (0%)	<0.001
	LGA	11 (21.6%)	0 (0%)	0.001
	SGA	3 (5.9%)	0 (0%)	0.095
	AGA	30 (58.8%)	46 (100%)	<0.001
	Macrosomia	7 (13.7%)	0 (0%)	0.009
APGAR score < 7	1 min after birth	4 (7.8%)	0 (0%)	0.05
	5 min after birth	4 (7.8%)	0 (0%)	0.05
Respiratory distress		16 (31.4%)	0 (0%)	<0.001
Hospitalization ≥ 24 h in NICU		5 (9.8%)	0 (0%)	<0.001
Stillbirth		0 (0%)	0 (0%)	
Neonatal death		0 (0%)	0 (0%)	
Neonatal hypoglycemia	Hypoglycemia 0–2 h	1 (1.96%)	0 (0%)	
	Hypoglycemia 3–47 h	2 (3.92%)	0 (0%)	
	Hypoglycemia 48–72 h	1 (1.96%)	0 (0%)	
Hyperbilirubinemia		4 (7.84%)	2 (4.34%)	
Congenital anomalies		1 (2%)	0 (0%)	

GDM, gestational diabetes mellitus; LGA, large for gestational age; SGA, small for gestational age; AGA, appropriate for gestational age; NICU, neonatal intensive care unit.
